# One-stage posterior debridement, bone grafting fusion, and mono-segment vs. short-segment fixation for single-segment lumbar spinal tuberculosis: minimum 5-year follow-up outcomes

**DOI:** 10.1186/s12891-020-3115-x

**Published:** 2020-02-07

**Authors:** Zheng Liu, Weiwei Li, Zhengchao Xu, Xiyang Wang, Hao Zeng

**Affiliations:** 1 Department of Spine Surgery, Xiangya Hospital, Central South University, 87#Xiangya Road, Changsha, 410008 Hunan People’s Republic of China; 2 Hunan Engineering Laboratory of Advanced Artificial Osteo-materials, Xiangya Hospital, Central South University, 87#Xiangya Road, Changsha, 410008 Hunan People’s Republic of China; 3 Department of Spinal Surgery, the First Affiliated Hospital of Guangxi Medical University, No. 6 Shuangyong Rd, Nanning, 530021 Guangxi China; 4 Guangxi Key Laboratory of Regenerative Medicine, Nanning, 530021 Guangxi China

**Keywords:** Posterior, Debridement, Fusion, Short-segment fixation, Mono-segment fixation, Single-segment lumbar spinal tuberculosis

## Abstract

**Background:**

To compare the clinical and radiological outcomes between posterior mono-segment and short-segment fixation combined with one-stage posterior debridement and bone grafting fusion in treating single-segment lumbar spinal tuberculosis (LSTB).

**Methods:**

Sixty-two patients with single-segment LSTB treated by a posterior-only approach were divided into two groups: short-segment fixation (Group A, *n* = 32) and mono-segment fixation (Group B, *n* = 30). The clinical and radiographic outcomes were analyzed and compared between the two groups.

**Results:**

The intraoperative bleeding volume, operation time, and hospitalization duration were lower in Group B than in Group A. All patients achieved the bony fusion criteria. The visual analog scale score, Japanese Orthopedic Association score, and Oswestry Disability Index were substantially improved 3 months postoperatively and at the last visit in both groups, with no significant difference between the two groups (*P* > 0.05). Kirkaldy–Willis functional evaluation at the final follow-up demonstrated that all patients in both groups achieved excellent or good results. The difference in the angle correction rate and correction loss between Groups A and B was not significant (*P* > 0.05).

**Conclusions:**

One-stage posterior debridement, bone grafting fusion, and mono-segment or short-segment fixation can provide satisfactory clinical and radiological outcomes. Mono-segment fixation is more suitable for the treatment of single-segment LSTB because the lumbar segments with normal motion can be preserved with less trauma, a shorter operation time, shorter hospitalization, and lower costs.

## Background

According to the latest report by the World Health Organization in 2018 [1], the number of new cases of tuberculosis (TB) worldwide exceeded 10 million and the estimated number of deaths reached 1.6 million in 2017. Among low- to middle-income countries, China ranked second among the 30 countries with the highest TB burden, second only to India.

Spinal TB (STB) is the most frequent and serious form of skeletal TB and can cause vertebral collapse, spinal deformity, neurological injury, and even paraplegia [2, 3]. Conservative treatment with anti-TB chemotherapy is the mainstay of STB therapy [4] and can yield good to excellent clinical outcomes in most patients; however, it cannot prevent kyphotic aggravation. Therefore, medical therapy is the fundamental means of curing musculoskeletal TB, and surgery is an adjunct to anti-TB chemotherapy. Surgery is performed not only to debride the lesion but also to decompress the spinal cord, restore normal spinal alignment, and reconstruct the spinal stability.

In 1911, Hibbs [5] and Albee [6] described posterior fusion as a surgical modality to hasten recovery from TB spondylitis. This approach was later abandoned because posterior fusion did not prevent progressive kyphosis, which could lead to paralysis. This may be due to the lack of strong internal fixation during the early stage of bone fusion, which may lead to poorly effective kyphosis correction and aggravation of kyphosis during the fusion period. A stable, rigid internal fixation system may prevent kyphosis progression and severe back pain caused by spinal instability. With the introduction of screw and rod fixation systems, increasingly more surgeons are adopting the posterior-only approach to treat STB [7–9]. In our previous study [2], we found that in treating thoracolumbar junction STB, long-segment internal fixation with a posterior-only approach prevailed over short-segment fixation in terms of kyphotic correction and maintenance of spinal stability, especially in the long-term prevention of angle loss.

However, because the lumbar area sustains the maximum spinal load and has a high range of motion, the fixation ranges differ between the lumbar and thoracolumbar spine. Because of the anatomic and biomechanical features of the lumbar region, the fixation range and surgical approach in treating lumbar STB (LSTB) are also controversial.

No comparative study has assessed LSTB treated by one-stage posterior debridement, fusion, and mono-segment vs. short-segment fixation. Therefore, the present study was performed to compare the clinical and radiological outcomes of posterior mono-segment and short-segment fixation combined with one-stage posterior debridement and bone grafting fusion in treating single-segment LSTB.

## Methods

### Basic information

In total, 76 patients with LSTB and a single-segment lesion who were admitted to our hospital from January 2008 to December 2013 were retrospectively reviewed.

The inclusion criteria were as follows: the main lesion involved a functional unit of the lumbar spine (L2–S1), mono-segment or short-segment fixation was performed by a posterior-only approach, pathological examination revealed a definitive diagnosis of TB, and the patient underwent a minimum 5-year follow-up.

The exclusion criteria were multi-level large paravertebral abscesses or huge iliopsoas abscesses; a history of lumbar surgery and/or other spinal diseases affecting the postoperative evaluation, such as adolescent scoliosis or ankylosing spondylitis; severe vertebral osteoporosis on radiographs; and absence of complete follow-up data for any reason, including loss to follow-up and death.

Based on our previous clinical experience, the indications for surgery were as follows [2]: significant or progressive radiculopathy or cauda equina syndrome due to compression from a TB lesion; severe bone destruction with spinal instability, pathological dislocation, or a developing spinal deformity; a vertebral TB lesion with formation of a large sequestrum or cavity; and persistent back pain resulting from an STB lesion after expectant treatment.

A definitive diagnosis was achieved by pathological examination of the surgically debrided specimen. Four patients in whom spinal TB could not be confirmed by histological examination were excluded from this study. Among the remaining patients, complete follow-up data were available for 62 (86%), and 10 (14%) were lost to follow-up.

According to the above screening criteria, 62 patients were included in this study and divided into 2 groups according to the fixation range. The short-segment fixation group (Group A) comprised 32 patients (18 male, 14 female) with a mean age of 39.5 ± 11.6 years (range, 19–58 years) who underwent one-stage posterior debridement, bone grafting fusion, and short-segment fixation (range to one upper and lower vertebra adjacent to the pathologic segment with/without fixation to the pathologic vertebrae). The mono-segment fixation group (Group B) comprised 30 patients (16 male, 14 female) with a mean age of 42.8 ± 8.8 years (range, 18–57 years) who underwent one-stage posterior debridement, bone grafting fusion, and mono-segment fixation (range limited to the pathologic segment).

Comprehensive assessment of clinical symptoms, laboratory results, and imaging findings was necessary to diagnose STB [10]. The main clinical symptoms included low back pain, radiating pain, dysesthesia, and dyskinesia of the lower limb. Some patients also had systemic toxicity symptoms such as fever, night sweats, anorexia, and weight loss. No patients had active lung TB or human immunodeficiency virus positivity. The active period of TB was confirmed by an increased erythrocyte sedimentation rate (ESR) and C-reactive protein (CRP) concentration in all patients. Imaging examinations were routinely performed. The lumbar imaging findings, such as collapsed vertebrae and necrotic discs, kyphotic deformity, cold abscess formation, and dura compression, were displayed on preoperative radiographs, computed tomography (CT) scans, or magnetic resonance images. The deformity angle was measured by drawing two lines on a lateral image: one on the superior surface of the uppermost-involved vertebra and the other through the inferior surface of lowermost-involved vertebra [11]. Pain severity was evaluated using a visual analog scale (VAS). The Japanese Orthopedic Association (JOA) score [12] and Oswestry Disability Index (ODI) [13] were applied to evaluate dysfunction and quality of life.

### Preoperative procedure

Preoperatively, all patients received standard anti-TB chemotherapy with HRZE for at least 2 weeks, including rifampicin (450 mg/day), isoniazid (300 mg/day), ethambutol (750 mg/day), and pyrazinamide (750 mg/day). Surgery was performed when the ESR and CRP concentration had significantly decreased and constitutional symptoms were obviously relieved.

### Surgical procedure

**Fig. 1:**
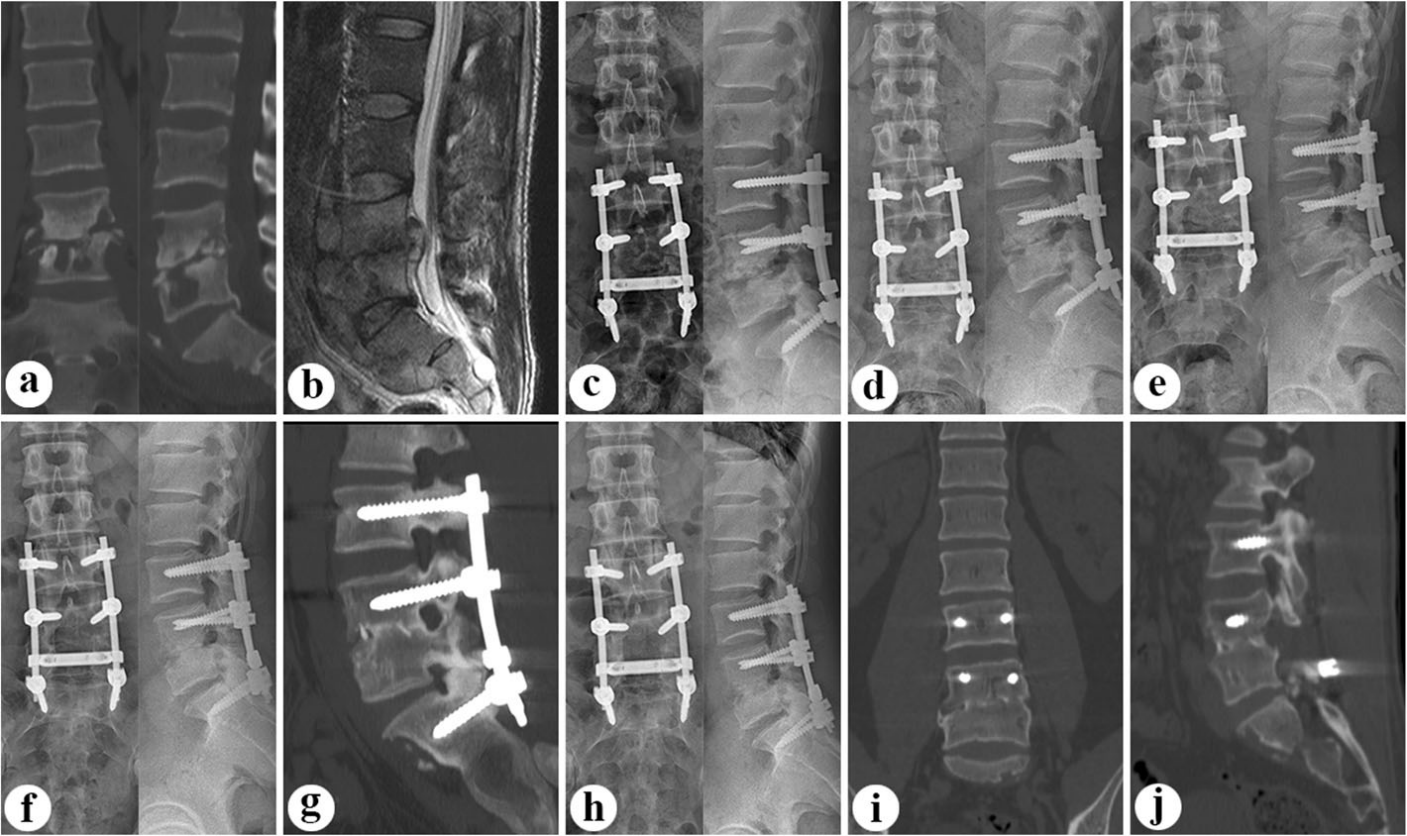
A patient with L4/5 lesion was performed by one-stage posterior debridement, bone grafting fusion, and short-segment fixation. The pre-operative images ((**a**) CT antero-posterior and lateral, (**b**) T-2 MRI lateral) showed severe bone destruction, paravertebral abscess formation, dural sac compression and a local lordotic angle (7.4°) at L4/5. Postoperative radiography (**c**) showed that fixation was in good position with an improved local lordotic angle (14.5°). (**d**) At 3-month after surgery, radiograph presented with interbody bone trabecular formation. During the follow-up, (**e**) 9-month, (**f-g**) 24-month after surgery, X-ray or CT displayed solid bone fusion without signs of fixation failure. (**i-j**) At the last visit (62 month after surgery), radiograph and CT illustrated strong bony fusion and no obvious correction angle loss (1°) with good fixation position

Patients in Group A (Fig. 1) underwent one-stage posterior debridement, bone grafting fusion, and short-segment fixation. Pedicle screws were implanted into the upper and lower vertebra adjacent to the pathologic segment. Whether the screws were fixed to the pathologic vertebrae was based on the extent of vertebral destruction. After installing a temporary internal fixation instrument on the opposite side to avoid nerve injury during contralateral focal debridement, hemi-laminectomy or complete laminectomy was performed on the more severely affected side. The removal of lesions, including TB granulation tissue, caseous necrotic material, sequestra, abscesses, and necrotic discs and endplates, was performed using curettes of different sizes and angles until scraping of the bone surface produced bleeding. By pressurized washing and negative-pressure suction (using a suitable flush tube with saline plunged into the depths of the lesion), all potential residual lesions were debrided as radically as possible.

Before tightening the rods, deformities were corrected by compressing and stretching the internal fixation instrument. An appropriately shaped block-sized allograft or autograft bone was inserted in the interbody to reconstruct the vertebral body. The space between the decorticated transverse processes was carpeted with autogenous or allogeneic particulate bone to promote bone fusion. Local anti-TB therapy with streptomycin (1 g) and isoniazid (0.3 g) was routinely administered in the surgical area. Drainage was routinely established before suturing the incision closed. Each patient’s debrided specimen underwent mycobacterial culture and histopathological examination.

**Fig. 2:**
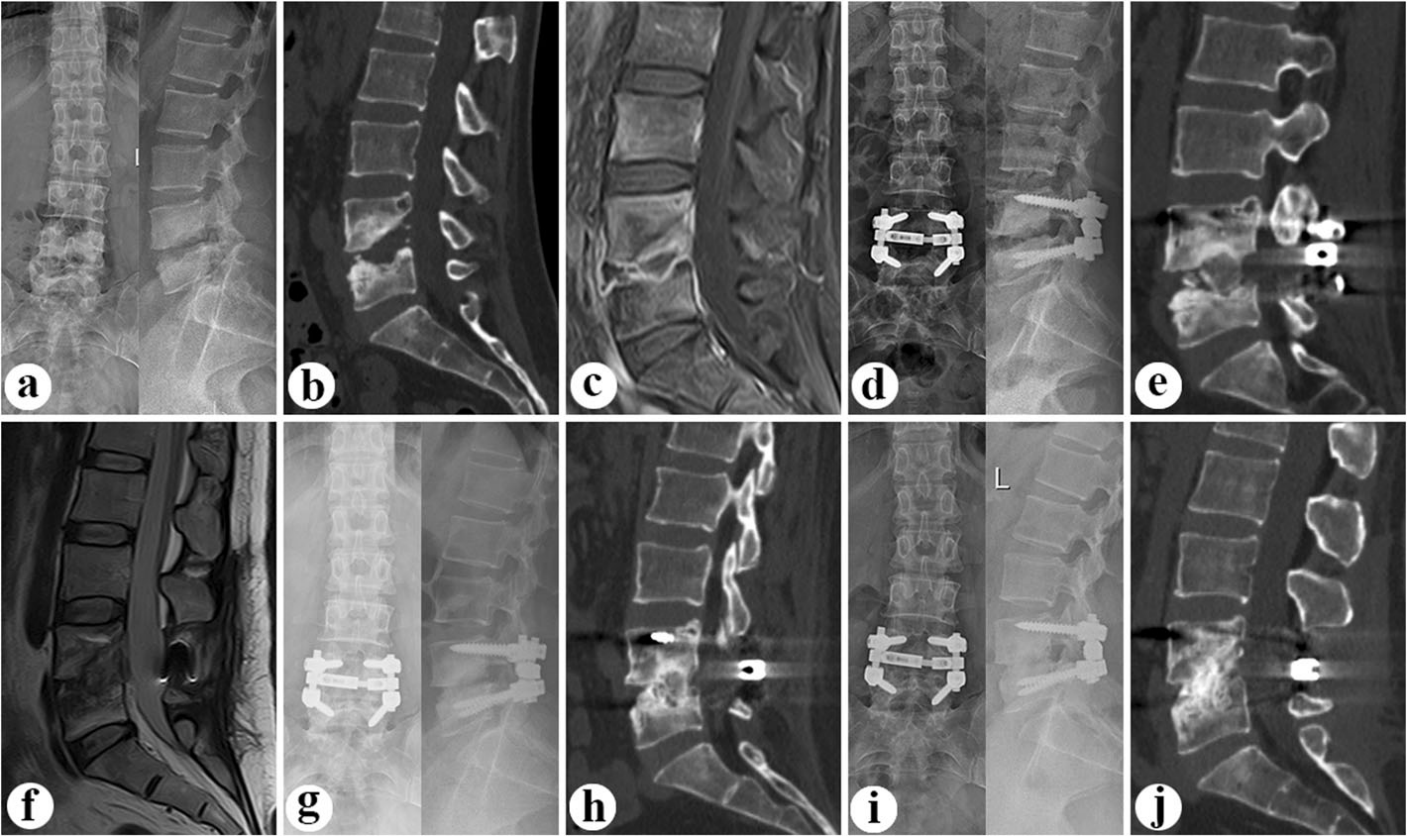
A patient with L4/5 lesion was performed by one-stage posterior debridement, bone grafting fusion, and mono-segment fixation. The pre-operative images ((**a**) plain antero-posterior and lateral, (**b**) CT lateral, (**c**) MRI lateral) illustrated severe vertebral destruction at L4/5 with a local lordotic angle (17°). Postoperative images (**d-f**) exhibited that internal fixation and implanted bone was in good position with an improved local lordotic angle (23.8°). (**g-h**) At 11-month after surgery, radiograph and CT showed strong bony fusion at interbody of L4/5. (**i-j**) At the final follow-up (64 month after surgery), X-ray and CT demonstrated strong bony fusion and no obvious correction angle loss (0.9°) with good fixation position

Patients in Group B (Fig. 2) underwent one-stage posterior debridement, bone grafting fusion, and mono-segment fixation. Pedicle screws were implanted and limited to the pathologic segment. Notably, the screws were located close to the endplates to maintain an adequate distance from the lesion, thus avoiding exposure of the screw after debridement. The other procedures were the same as in Group A.

All operations were performed by the same group of surgeons, and similar implants were used in all procedures.

### Postoperative procedure

The postoperative drainage tube was retained until the drainage flow was < 30 ml/day. Intravenous anti-infection and nutritional agents were routinely administered. A standard anti-TB regimen with HRZE was administered for 3 months. The pyrazinamide was then discontinued, and the HRE chemotherapy was continued for 9 to 15 months. Because of the potential adverse effects of anti-TB drugs, hepatic and renal function was regularly evaluated. Two weeks after surgery, gradual walking was allowed with the aid of a spinal orthosis. During the next 3 to 6 months, the orthosis was removed when interbody bony callus formation was evident on imaging examinations.

### Follow-up evaluation

The ESR and CRP concentration were measured to assess the activity of the TB lesion during the perioperative period and at 3 months postoperatively. During the follow-up, the positions of the graft and instrumentation were investigated by routine radiography or CT, which was also performed to evaluate the graft fusion status during follow-up according to the modified radiological criteria established by Lee et al. [14]. The local deformity angle, JOA score, ODI, and VAS score were recorded preoperatively, postoperatively, and at the final follow-up. At the last visit, the Kirkaldy–Willis functional outcome [15] was used to evaluate the patients’ living and working conditions.

### Statistical analysis

The statistical analysis in this study was performed by SPSS 24.0 statistical software (IBM Corp., Armonk, NY, USA). Student’s *t* test was used to compare the clinical data between the two groups. A paired *t*-test was applied to compare the changes in indices in each group preoperatively, postoperatively, and during follow-up. Any discrepancy in the normal distribution was analyzed using the rank sum test. A *P* value of < 0.05 was considered statistically significant.

## Results

### Outcomes

All patients experienced significant improvement in their clinical symptoms after surgery and achieved complete cure during follow-up. The clinical data of the patients in the two groups are shown in Table 1. The intraoperative blood loss volume, operation time, and hospitalization duration were lower in Group B than A (*P* < 0.05). The mean 95% confidence intervals were 24.955, 98.538, and 2.982, respectively.

**Table 1:** The clinical data of patients in two groups

	Group A (*N* = 32)	Group B (*N* = 30)	*P*-value	Mean (95% CI)
Operation time (min)	162.4 ± 21.4	137.4 ± 22.6	0.000 < 0.05	24.955
Blood loss (ml)	763.9 ± 85.9	665.3 ± 111.9	0.000 < 0.05	98.538
Hospitalization (days)	16.5 ± 2.2	13.6 ± 2.3	0.000 < 0.05	2.982
Duration of follow-up (months)	65.2 ± 3.7	66.1 ± 4.5	0.411	−0.873
Fusion time (months)	7.9 ± 1.0	8.2 ± 1.4	0.296	−0.330
ESR (mm/h)				
Pre	68.0 ± 7.1	67.1 ± 7.2	0.613	0.933
TMP	9.4 ± 2.4	9.9 ± 2.7	0.498	−0.447
CRP (mg/l)				
Pre	41.1 ± 11.9	42.3 ± 8.4	0.637	−1.238
TMP	5.6 ± 1.7	6.0 ± 1.7	0.341	−0.420

*Pre* preoperative, *TMP* three months postoperative, *FFU* final follow-up, *CI* confidence intervals

The differences in the mean follow-up duration and average fusion time were not significant between the two groups (*P* > 0.05). All patients achieved the definitive bony fusion criteria [14] based on radiographic and/or CT assessment. The ESR and CRP level decreased to the reference range in both groups at 3 months postoperatively.

The results of the pain and dysfunction evaluation are presented in Table 2**.** The VAS score, JOA score, and ODI showed obvious improvement at 3 months postoperatively and the last visit in both groups (*P* < 0.05), and there was no significant difference between the two groups (*P* > 0.05). The Kirkaldy–Willis functional evaluation at the final follow-up demonstrated that all patients in both groups achieved excellent or good results. At the last visit, all patients reported a normal return to their life and work.

**Table 2:** The evaluation outcomes of pain and dysfunction

Group (*n*)	VAS			JOA			ODI			Kirkaldy–Willis criteria			
	Pre	TMP	FFU	Pre	TMP	FFU	Pre	TMP	FFU	E	G	F	P
A (32)	7.6 ± 0.9	2.5 ± 0.6	0.9 ± 0.6	12.5 ± 2.7	17.3 ± 3.0	25.4 ± 2.5	37.9 ± 4.8	17.6 ± 3.5	7.1 ± 2.0	20	12	0	0
B (30)	7.6 ± 1.1	2.6 ± 0.6	0.9 ± 0.6	12.9 ± 2.9	17.5 ± 3.3	25.9 ± 2.6	36.8 ± 5.2	17.1 ± 3.3	7.4 ± 1.7	22	8	0	0
*P*-value	0.942	0.591	0.641	0.530	0.736	0.404	0.395	0.580	0.574				
Mean (95% CI)	−0.019	−0.083	0.069	−0.449	− 0.275	− 0.545	1.103	0.481	−0.271				

*Pre* preoperative, *TMP* three months postoperative, *FFU* final follow-up, *E* excellent, *G* good, *F* fair, *P* poor, *CI* confidence intervals

Comparison of the local lumbar deformity angle in Groups A and B is shown in Table 3**.** According to radiographic or CT measurement, 34 patients (A_*k*_ and B_*k*_) had a local lumbar kyphotic angle while 28 patients (A_*l*_ and B_*l*_) had a local lumbar lordotic angle. Groups A and B showed no significant differences in the local lumbar deformity angle preoperatively, immediately postoperatively, or at the last follow-up (*P* > 0.05). In both groups, the local lumbar deformity angle was significantly improved compared with the preoperative angle (*P* < 0.05). The difference in the angle correction rate between Groups A and B was not significant (*P* > 0.05). At the last visit, the mean correction loss was 0.8° ± 0.3° and 0.9° ± 0.3° in Group A_*k*_ and B_*k*_ (*P* > 0.05) and 0.7° ± 0.3°and 0.6° ± 0.2° in Group A_*l*_ and B_*l*_ (*P* > 0.05), respectively.

**Table 3:** Comparison of the local lumbar deformity angle

Group	*n*	Kyphosis angle (°)			Angle correction		Correction loss (°)
		Pre	Post	FFU	Post (°)	Rate (%)	
A_*k*_	20	18.4 ± 6.8	5.4 ± 1.7	6.2 ± 1.7	12.8 ± 5.7	68.1 ± 9.7	0.8 ± 0.3
B_*k*_	14	18.0 ± 6.7	5.6 ± 1.6	6.5 ± 1.6	12.4 ± 5.4	66.9 ± 6.2	0.9 ± 0.3
*P*- value		*P* = 0.866	*P* = 0.702	*P* = 0.699	*P* = 0.810	*P* = 0.699	*P* = 0.776
Mean (95% CI)		0.404	−0.219	−0.246	0.473	1.149	−0.026
		Lordotic angle(°)					
		Pre	Post	FFU			
A_*l*_	12	10.0 ± 5.5	18.7 ± 4.9	18.0 ± 4.9	8.6 ± 2.5	46.3 ± 22.4	0.7 ± 0.3
B_*l*_	16	10.5 ± 5.3	19.0 ± 3.8	18.5 ± 3.8	8.5 ± 2.2	48.1 ± 19.2	0.6 ± 0.2
*P*- value		*P* = 0.815	*P* = 0.822	*P* = 0.778	*P* = 0.909	*P* = 0.823	*P* = 0.289
Mean (95% CI)		−0.488	−0.375	−0.477	0.102	−1.783	0.102

*Pre* preoperative, *post* postoperative immediately, *FFU* final follow-up, *CI* confidence intervals

A_*k*_ and B_*k*_: the cases with a local lumbar kyphosis angle in lesions

A_*l*_ and B_*l*_: the cases with a local lumbar lordotic angle in lesions

### Complications

Postoperative complications occurred in both groups. Two patients in Group A and one patient in Group B developed a superficial wound infection that healed with anti-infection agents and wound dressings. One patient in each group developed cerebrospinal fluid leakage, which was cured by leaving the drainage tube in place longer and increasing the fluid therapy. One patient in Group B developed liver function damage induced by anti-TB drugs; this patient was treated with modified chemotherapy combined with hepatic protection drugs. No complications related to internal fixation occurred in either group during follow-up.

## Discussion

### Characteristics of LSTB

The lumbar region sustains the largest load and exhibits the greatest mobility among all spinal regions. Because of the large pressure and shear force on the lumbar segments, lumbar stability is maintained by the combined effects of the vertebrae, intervertebral discs, rich muscle groups, and tough ligaments. The local anatomical structures adjacent to the lumbar region are complex and include major blood vessels, nerves, and the ureters.

LSTB mainly involves the anterior and middle lumbar columns, potentially leading to vertebral destruction and collapse, changes in the physiological lordosis and load biomechanics, kyphosis deformity, and protrusion of the pathological tissues into the vertebral canal, seriously affecting patients’ health and quality of life. LSTB usually presents with low back pain with or without radicular leg pain and neurologic deficits secondary to compression of the cauda equina and nerve roots.

### Importance of posterior-only approach

Surgical procedures in the posterior-only approach, including lesion debridement and bone grafting and fixation, can be accomplished simultaneously using one incision without changing the position; thus, this approach is much less invasive than others. Additionally, the posterior-only approach is familiar to spinal surgeons and avoids possible injury to the large blood vessels, nerves, or other anatomical structures. Furthermore, internal fixation in the posterior-only approach is more effective than that in the anterior approach with respect to kyphotic correction and the maintenance of correction [16].

However, many surgeons are concerned about the potential increase in spinal instability caused by damage to the posterior column. In our experience, the strong three-column fixation can effectively maintain short-term postoperative spinal stability. Furthermore, strong bony fusion can be obtained by the combination of interbody bone grafting and lateral bone grafting or posterior lamina reconstruction, maintaining long-term spinal stability.

The concentration of TB lesions mainly in the anterior column has given rise to controversy regarding whether the posterior approach can completely achieve focal debridement. Indeed, the posterior-only approach offers no advantage with respect to debridement. However, removal of the lamina and facet joints with moderate stretching of the nerve roots and dura mater can provide adequate surgical space in which 360° lesion debridement under direct vision can be achieved. Moreover, subsequent procedures, including saline irrigation at the lesion site with pressurized washing and negative-pressure suction and postoperative postural drainage, can effectively drain pus and eliminate residual lesions [17]. Furthermore, the cleared lesion can facilitate the penetration of anti-TB drugs, improving the efficacy of local anti-TB drugs intraoperatively and systemic anti-TB drugs postoperatively and resulting in cure through spontaneous fusion in STB lesions. Therefore, complete debridement need not be overemphasized [18].

### Choice of fixation range

The choice of the fixed segment range is the focus of long-standing debate in lumbar fixation. In this study, the fixed range of mono-segment fixation was limited to the pathologic segment. Short-segment fixation is defined as limitation of the fixed range to one upper and lower vertebra adjacent to the pathologic segment, with or without inclusion of the pathologic segment according the extent of the vertebral destruction.

Short-segment fixation [19] provides strong fixation and deformity correction and is still applied by most surgeons for treatment of single-segment LSTB. The increase in the fixed segment range can distribute the longitudinal stress of the spine to longer segments, which can significantly maintain the spinal stability, improve the vertebral body height, and prevent loss of the correction angle. However, short-segment fixation also sacrifices the motion of the two normal segments, affecting the activity of the lumbar spine in the long term and leading to aggravation of adjacent segment degeneration (ASD), which may ultimately induce ASD-related diseases [20]. One study [21] showed that longer fixed segments result in greater stress concentration and exert greater loads on adjacent segments, which may accelerate ASD and cause lower back pain, pseudarthrosis, and implant rupture.

Because of the anatomical features and high requirement of activity in the lumbar region, preserving segments with normal motion is important to ensure high long-term quality of life. Thus, mono-segment rather than short-segment fixation is superior for LSTB. However, many surgeons are concerned that mono-segment fixation cannot meet the requirements of reconstruction stability in treating STB. In this study, we compared the clinical and radiological outcomes of posterior short-segment versus mono-segment fixation combined with one-stage posterior debridement and bone grafting fusion in treating single-segment LSTB.

Maida et al. [22] compared the clinical and radiological outcomes of mono-segment fixation with bi-segment fixation in treating thoracolumbar spine fractures. They found no statistically significant difference in vertebral body height restoration or correction of the kyphotic deformity between the two groups, confirming the validity of mono-segment fixation. Wei et al. [23] demonstrated that both mono-segment fixation and short-segment fixation are effective and reliable for thoracolumbar burst fractures. Mono-segment fixation significantly shortened the operative time and decreased the amount of blood loss, thus offering better clinical results. Li et al. [24] reported that the mean postoperative VAS score and vertebral kyphotic angle were similar in the mono-segment fixation group and short-segment fixation group. These clinical studies have demonstrated that mono-segment fixation can meet the stability requirements necessary for spinal fracture reconstruction, which has also been confirmed in biomechanical experiments and finite element analysis [25, 26].

Various degrees of reactive new bone formation can be seen in the involved vertebrae in most patients with STB. The vertebrae involved by *Mycobacterium tuberculosis* form sclerotic bone walls, resulting in abnormally high bone density of the pathological vertebrae. This pathologic feature of STB brings stronger holding forces of the pedicle screws to the involved vertebrae than in spinal fracture, making mono-segment fixation more feasible for treating STB than spinal fractures. Wang et al. [27] reported that after bone fusion, mono-segment fixation was effective in restoring and maintaining spinal stability and retained normal-motion segments more than did short-segment fixation.

In the present study, the mean local angle correction rate and correction loss were 66.9% ± 6.2% and 0.9° ± 0.3° in Group B_*k*_ and 48.1% ± 19.2% and 0.6° ± 0.2° in Group B_*l*_, respectively. There were no differences in the deformity angle correction rate or correction loss between Groups A and B (*P* > 0.05). These findings indicate that mono-segment fixation can achieve satisfactory effectiveness in restoring and maintaining spinal stability, similar to short-segment fixation.

Mono-segment fixation has several advantages in treating single-segment LSTB. Above all, the lumbar segments with normal motion can be retained, which may slow the degeneration of adjacent segments to some extent. Additionally, the surgical field of exposure in mono-segment fixation is relatively smaller and less invasive. Furthermore, the need for fewer fixation materials reduces the operation time and hospitalization costs, which can ease the burden on patients and may be more suitable for patients in developing countries and poorer areas. In the present study, the mean operative time, amount of blood loss, and hospitalization duration in Group B were 137.4 ± 22.6 min, 665.3 ± 111.9 ml, and 13.6 ± 2.3 days respectively, all of which were lower than those in Group A (*P* < 0.05).

The application of mono-segment fixation in treating single-segment LSTB also has some limitations. Because of the irregular destruction created by LSTB, the degree of vertebral destruction varies among different patients. The choice for mono-segment fixation should be based on the extent of vertebral destruction. Wang et al. [28] considered that the remaining one- to two-thirds of the vertebral height is enough to accommodate a conventional transpedicular screw after debridement, which is consistent with our experience. In addition, the structural integrity, including the pedicle in the diseased vertebrae, the upper endplate of the upper pathologic vertebra, and the lower endplate of the lower pathologic vertebra, are important for screw implantation.

Therefore, mono-segment fixation can be chosen when the following two conditions are fulfilled: a major lesion involving one motion unit of the lumbar spine is present, and enough space is available for screw implantation in the vertebrae adjacent to the lesion (more than one-third the vertebral height, with structural integrity of the pedicle and endplates in the pathologic vertebrae). Furthermore, mono-segment fixation is unsuitable for patients with severe kyphosis deformity or osteoporosis. Short-segment fixation is more suitable when the pathologic vertebral height is less than one-third.

This study has two main limitations. First, its retrospective nature may have resulted in biased outcomes. Second, the sample size was relatively small and the follow-up duration was relatively short. Therefore, prospective studies with larger samples and longer follow-up periods are needed.

## Conclusions

Either one-stage posterior debridement, bone grafting fusion, and mono-segment or short-segment fixation can achieve satisfactory clinical and radiological outcomes. However, mono-segment fixation is more suitable in treating single-segment LSTB because the lumbar segments with normal motion can be preserved with less trauma, a shorter operation time, shorter hospitalization, and lower costs.

## Abbreviations

CRP: C-reactive protein
CT: Computed tomography
ESR: Erythrocyte sedimentation rate
JOA: Japanese Orthopaedic Association
LSTB: Lumbar spinal tuberculosis
ODI: Oswestry-Disability index
STB: Spinal tuberculosis
TB: Tuberculosis
VAS: Visual analogue scale

## Availability of data and material

The datasets generated and/or analyzed during the current study are available from the corresponding author on reasonable request.
